# Profiling the BLAST bioinformatics application for load balancing on high-performance computing clusters

**DOI:** 10.1186/s12859-022-05029-7

**Published:** 2022-12-16

**Authors:** Trinity Cheng, Pei-Ju Chin, Kenny Cha, Nicholas Petrick, Mike Mikailov

**Affiliations:** 1 Center for Devices and Radiological Health, U.S. Food and Drug Administration, Silver Spring, MD 20993 USA; 2 Department of Biomedical Engineering, Whiting School of Engineering, Johns Hopkins University, Baltimore, MD 21218 USA; 3 Center for Biologics Evaluation and Research, U.S. Food and Drug Administration, Silver Spring, MD 20993 USA

**Keywords:** BLAST, High performance computing (HPC), Load balancing, Parallelization, Performance model, Profiling

## Abstract

**Background:**

The Basic Local Alignment Search Tool (BLAST) is a suite of commonly used algorithms for identifying matches between biological sequences. The user supplies a database file and query file of sequences for BLAST to find identical sequences between the two. The typical millions of database and query sequences make BLAST computationally challenging but also well suited for parallelization on high-performance computing clusters. The efficacy of parallelization depends on the data partitioning, where the optimal data partitioning relies on an accurate performance model. In previous studies, a BLAST job was sped up by 27 times by partitioning the database and query among thousands of processor nodes. However, the optimality of the partitioning method was not studied. Unlike BLAST performance models proposed in the literature that usually have problem size and hardware configuration as the only variables, the execution time of a BLAST job is a function of database size, query size, and hardware capability. In this work, the nucleotide BLAST application BLASTN was profiled using three methods: shell-level profiling with the Unix “time” command, code-level profiling with the built-in “profiler” module, and system-level profiling with the Unix “gprof” program. The runtimes were measured for six node types, using six different database files and 15 query files, on a heterogeneous HPC cluster with 500+ nodes. The empirical measurement data were fitted with quadratic functions to develop performance models that were used to guide the data parallelization for BLASTN jobs.

**Results:**

Profiling results showed that BLASTN contains more than 34,500 different functions, but a single function, RunMTBySplitDB, takes 99.12% of the total runtime. Among its 53 child functions, five core functions were identified to make up 92.12% of the overall BLASTN runtime. Based on the performance models, static load balancing algorithms can be applied to the BLASTN input data to minimize the runtime of the longest job on an HPC cluster. Four test cases being run on homogeneous and heterogeneous clusters were tested. Experiment results showed that the runtime can be reduced by 81% on a homogeneous cluster and by 20% on a heterogeneous cluster by re-distributing the workload.

**Discussion:**

Optimal data partitioning can improve BLASTN’s overall runtime 5.4-fold in comparison with dividing the database and query into the same number of fragments. The proposed methodology can be used in the other applications in the BLAST+ suite or any other application as long as source code is available.

## Background

The Basic Local Alignment Search Tool (BLAST) is a computer algorithm developed and maintained by the National Institutes of Health, National Center for Biotechnology Information (NIH/NCBI) for identifying regions of identity and statistical importance between biological sequences such as nucleotides or proteins. BLAST is used in different areas of bioinformatics including nucleotide sequence mapping, genomic research, species identification, etc. Since its development in 1990, BLAST has become one of the most widely used bioinformatics applications, with the original publication cited more than 94 thousand times [1].

BLAST takes two user-input files, *database* and *query*, that usually consist of millions of biological sequences. A biological sequence is a single, continuous molecule: either a nucleic acid composed of nucleotides or a peptide composed of amino acids. BLAST can compare these biological sequences to the existing database and calculate the statistical significance of the matches. A match between two sequences is found when two sub-sequences are identical, or similar when some unmatched characters or gaps are omitted.

**Fig. 1:**
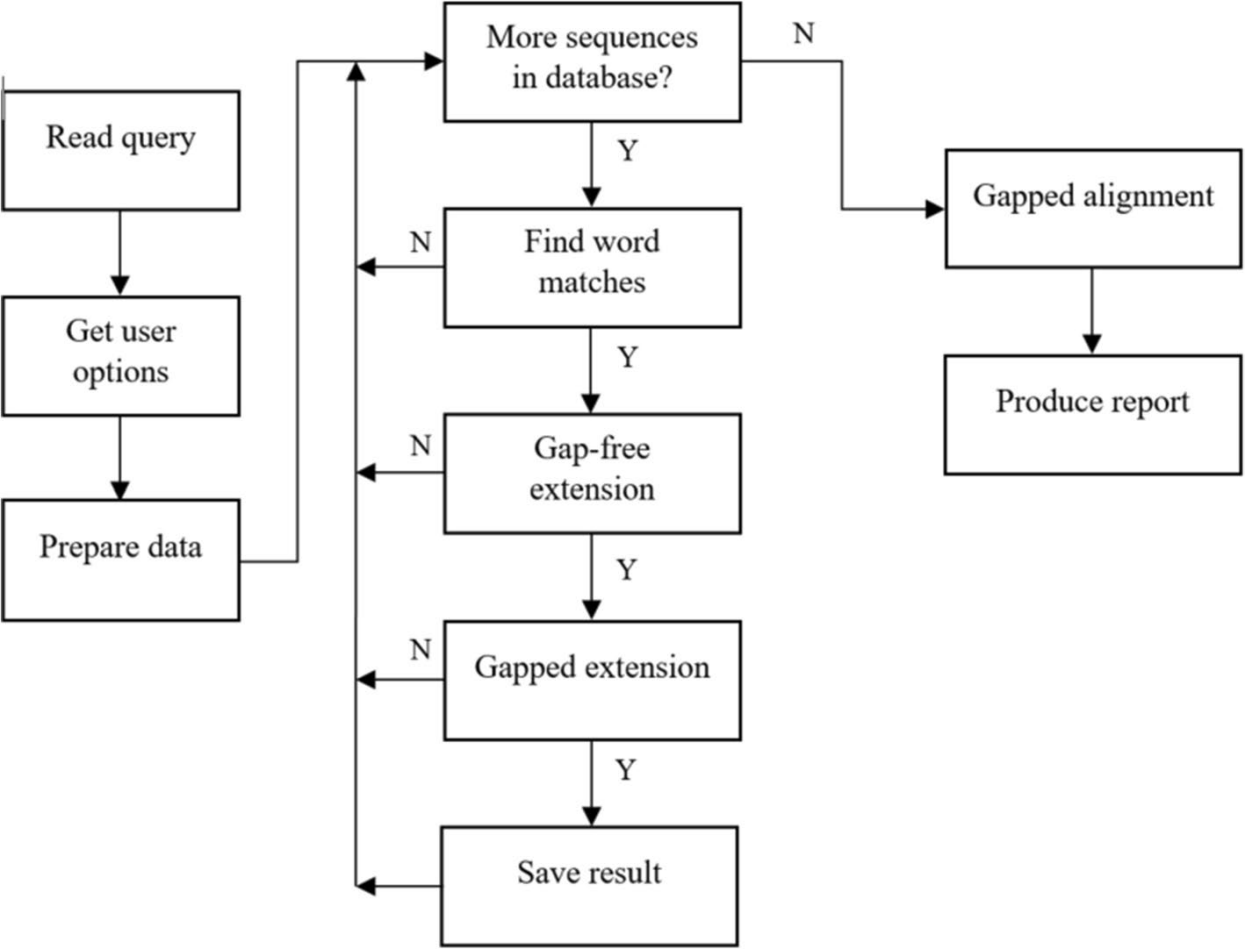
Outline of the BLAST process [3]

The NIH/NCBI command-line BLAST package “BLAST+” includes database manipulation tools, core BLAST search programs, specialized protein search programs, etc. [2]. The five core BLAST search programs each specializes in a different type of search: BLASTN (comparison of nucleotide database and nucleotide query), BLASTP (comparison of peptide database and peptide query), BLASTX (comparison of nucleotide database and translated nucleotide query), TBLASTN (comparison of translated nucleotide database with nucleotide query), and TBLASTP (comparison of translated peptide database with peptide query). The BLAST+ package is distributed in C++ source code as well as installable executables for the Linux, Windows, and MacOS platforms with detailed documentation [3]. The basic BLAST process is summarized in Fig. 1 and described further in the Results section.

Besides NIH/NCBI, researchers have developed various parallel implementations of the BLAST algorithm. For example, mpiBLAST is an open-source implementation of BLAST which uses Message Passing Interface (MPI) to split a BLAST database such that each node processes only a portion of the database. Database segmentation reduces disk Input/Output (I/O) by containing smaller data in the memory, resulting in near linear speedup in most cases [4]. ScalaBLAST can parallelize BLAST across more than 16,000 processors by dynamically scheduling BLAST calculations across processor pairs for fault-resilient speed-up [5]. miBLAST locates, then expands, word hits between the database and query to process batch workloads efficiently [6]. Divide and Conquer BLAST (DCBLAST) is an HPC computing wrapper that automatically divides BLAST queries among processors for speed-up [7].

Non-general purpose processors are also used in BLAST applications. A customized Field-Programmable Gate Array (FPGA) was designed to accommodate BLAST algorithm with programmable circuits [8]. Graphics processing unit (GPU)-accelerated versions of BLAST, such as CUDA-BLASTP [9], GPU-BLAST [10], and G-BLASTN [11], have also been developed to swarm smaller execution units to GPUs to process the smaller tasks simultaneously.

In contrast, parallelization using data partitioning provides a simple way to immediately utilize the available resources on an HPC cluster to speed up the latest version of the original BLAST+ without modifying the algorithm. For example, the “dual segmentation” method [12] divides the database and query into *m* and *n* subsets, respectively, on a cluster with $m \times n$ nodes. The $m \times n$ pairs of database-query subsets are then processed in parallel using $m \times n$ nodes. The only required modification to the source code is a global variable defined by the option “-dbseqnum” which specifies the effective number of sequences in the database. This value is important for calculating the *expect value*, or *E*-value, which is a measure of the statistical significance of the matches found. Experiment results showed reduction in runtime from 27 days to less than one day on a homogeneous HPC cluster with 500+ nodes [12]. However, selection of the optimal *m* and *n* values was not explored.

When a BLAST job is processed concurrently on multiple nodes on an HPC cluster, the turnaround time is determined by the longest sub-job among all nodes. The runtime required by each sub-job may differ because of the asymmetry of the computing nodes. For example, the computing nodes on a heterogeneous HPC cluster may not have the same hardware components. Even on a homogeneous HPC cluster, different computing nodes may perform differently because of the dynamic workload imposed by other jobs. Therefore, if a BLAST job is partitioned evenly among nodes without considering their performance differences, the nodes with more workload or less computing power will finish the sub-job late and prolong the turnaround time of the whole job.

The optimal load balancing configuration can be calculated by minimizing the following cost function [13]:

1 $$\begin{aligned} \max _i \{ e_i \} \, \end{aligned}$$

where $e_i$ is the runtime of a sub-job *i* and the overall runtime is determined by the slowest sub-job. It should be noted that BLAST runtime can vary based on sequence content [18]. Therefore, the runtime for a sub-job will vary based on the given database and query inputs. The runtime of each sub-job is estimated by performance models, and the objective is to minimize the runtime of the slowest sub-job. The constant performance model (CPM) [14] assumes that the speed, the amount of processed data per unit time, of each processor is a constant $s_i$ so the execution time for problem size $D_i$ is

2 $$\begin{aligned} e_i = \frac{D_i}{s_i}. \end{aligned}$$

The functional performance model (FPM) [13] [15] [16] can be used when the processor speed is a function of the problem size:

3 $$\begin{aligned} e_i = \frac{D_i}{s_i(D_i)}. \end{aligned}$$

Both CPM and FPM assume the problem size to be one-dimensional, which is not suitable for BLAST applications that take two input files. For example, the scalability of BLASTP was investigated separately by splitting database vs. splitting query [17]. In this study, the database is divided into *m* pieces, each of size $D_i$, where $1 \le i \le m$ and the query is divided into *n* pieces, each of size $Q_j$, where $1 \le j \le n$. Therefore, the BLAST data size for each sub-job is represented by $(D_i,Q_j)$. Since each unique pair of $D_i$ and $Q_j$ needs to be computed, there are $m \times n$ sub-jobs in total. The runtime for each sub-job on node type *k* can thus be denoted as

4 $$\begin{aligned} e_{i,j} = T_k(D_i,Q_j), \end{aligned}$$

where *T* is the estimated runtime for sub-job of size $(D_i,Q_j)$, i.e., the performance model. The cost function for a BLAST application becomes

5 $$\begin{aligned} \max _{i,j} \{ e_{i,j}\}. \end{aligned}$$

The solution to the load balancing problem is the database and query partitioning (*i*, *j*) which will minimize overall runtime.

In this study, the BLASTN application was profiled with various database sizes, query sizes, and node types to obtain the performance models. The goal was to improve BLASTN performance by load-balancing a heterogeneous HPC cluster.

## Methods

### Source code

The subject application of this study was BLASTN version 2.12.0. Two versions of BLASTN executables were built: the default configuration and the “with-profiling” configuration. The dbseqnum variable was added to the source code before compilation [12]. Multithreading was controlled by specifying “-num_threads=1.”

### Input data – database and query

A 2.4 GB test “nt” (nucleotide) database of 523,449 sequences downloaded from NCBI on June 16, 2017 was used in this study following the experiment set-up used in [12]. The database was truncated to generate six smaller databases as listed in Eq. 6 (units in number of sequences). The database sizes will be referenced as 8k, 16k, 32k, 64k, 130k, and 260k in the remainder of the paper. These database subsets ranged from 12 MB to 493 MB in file size. Some database sequences were greater than 10,000 base pairs in length.

6 $$\begin{aligned} D_i \in \{ 8179, 16358, 32716, 65432, 130862, 261725\} \end{aligned}$$

In order to test the reproducibility of our method with different datasets and study the impact of query content on BLASTN performance, two queries were prepared. Query A was a 15 GB query of 73,102,023 metagenomic short-read sequences from bacteria, which was selected following the experiment set-up used in [12]. Query B was a 20.7 GB query of 332,046,784 metagenomic short-read sequences originating from a published spiking study, with the viruses spiked into human HeLa cell background [19].

Both queries were truncated to generate 15 smaller queries each as listed in Eq. 7 (units in number of sequences). The query sizes will be referenced as 18k, 36k, 54k, 72k, 90k, 110k, 120k, 140k, 290k, 380k, 480k, 570k, 700k, 820k, and 1100k in the remainder of the paper. Query A subsets ranged from 3 MB to 233 MB in size, and the query sequences ranged from 90 to 150 base pairs in length. Query B subsets ranged from 3 MB to 198 MB in size, while the sequences were all 101 base pairs in length.

7 $$\begin{aligned} \begin{aligned} Q_j \in \{17848, 35695, 53555, 71389, 89258, 107188, 124961, 142778, \\ 285555, 393022, 480935, 571110, 696210, 821372, 1142220\} \end{aligned} \end{aligned}$$

### HPC environment

Experiments in this study were run on a U.S. Food and Drug Administration (FDA) Center for Devices and Radiological Health (CDRH) High Performance Computing (HPC) cluster, which consists of 500+ computing nodes in different hardware configurations, allowing for massive parallelization across thousands of cores. The cluster supports interconnection among nodes by 1 Gbps Ethernet, 10 Gbps Ethernet, and 100 Gbps Infiniband networks. Six different node types were tested in the experiments. Storage to the nodes is provided via DataDirect Networks storage cluster using IBM Spectrum Scale file system via 56 Gbps Infiniband connection. Table 1 lists the key hardware specifications. BLASTN was run with one, two, four, and eight threads to understand how multithreading impacts hardware-level parallelization performance.

**Table 1:** Hardware specifications of the node types tested in the experiments.

Node type	CPU model	Release year	Clock (GHz)	Chipset	#CPU /node	#Core /CPU	L2 cache /core (MB)	L3 cache /node (MB)	Memory (GB)	Network interface (ethernet/ infiniband)
I	Gold 6226	2019	2.70	C621	2	12	1	19.25	768	10G Eth
II	X5550	2009	2.67	5550	2	4	0.25	8	48	1G Eth
III	E7-4870	2011	2.40	C602J	4	10	0.25	30	256	1G Eth
IV	E5-2650 v4	2016	2.60	C612	2	12	0.25	30	512	10G Eth
V	E5-4627 v4	2016	2.60	C612	4	10	0.25	25	2048	10G Eth
VI	Silver 4116	2017	2.10	C622	2	12	1	16.5	512	100G IB

Node types are numbered based on their organization within the FDA HPC cluster. Storage to the nodes is provided via DDN storage cluster using IBM Spectrum Scale file system via 56 Gbps Infiniband connection

### Profiling

Three profiling techniques at different levels were used to study the factors that impact the BLASTN performance.

#### Unix time (shell-level)

The Unix “time (1)” is a shell command for measuring the time used by an application. It provides three different measures of execution time – real, user, and system. The “real” time is the total wall clock time, from the start to the end of an application. The “user” and “sys” times are the time spent in the user and kernel modes, respectively. Intuitively, the sum of the “user” and “sys” times should equal to the “real” time. However, “real” time will be greater than the sum of “user” and “sys” if the application is delayed by workloads from other processes, and vice versa if the application is accelerated by multithreading. The time command does not require the application source code. However, it only provides overall timing without a finer breakdown at the function level.

#### BLAST_PROF (code-level)

BLAST+ includes a built-in profiling module implemented in rtprofile.cpp. This module provides stopwatches for programmers to measure the “real” time spent by a section of code. A programmer can define a stopwatch and add start/stop points in any of the C++ programs in BLAST, then recompile the package. Each run generates a unique text file named after its process identification number (PID). Profiling can be switched on or off by the end user through the environmental variable “BLASTAPI_PROFILE_LOG” without recompiling the source code. Although this profiling method does not cost extra execution time running in the operating system kernel mode like “gprof” does, it does require the source code, recompilation, and knowledge of the BLAST algorithms.

#### Unix gprof (system-level)

The “gprof” program is a standard profiling tool provided by Unix [20]. To use this feature, the source code must be compiled with the profiling option “-pg” such that extra profiling code for each function is inserted into the executable. Running the profile-version executable will generate a trace file as “gmon.out” that includes the timing information of all function calls. The binary file “gmon.out” can then be translated by the “gprof” program to generate a human-readable text file. The “gmon.out” output contains two parts: a flat list of all functions sorted by execution time and a call graph showing the caller/callee relationships of all functions.

In BLAST+, the gprof profiling option can be enabled by the “–with-profiling” option in the configuration stage before compilation.

Since BLASTN contains more than 34,500 functions, which equate to the number of nodes in the call graph, two additional tools were used to visualize the colossal call graph. The “gprof2dot” program was used to convert the call graph into a mathematical graph in the “dot” file format. Then the “graphviz” program was used to render the mathematical graph into an image in the SVG file format. While “gprof” can collect very fine timing information, the stages of compilation, execution, and analysis are time-consuming. Running the profile-version BLASTN executable takes approximately five times longer than the original, and running “gprof” can add more than 45 additional minutes.

### Performance modeling

The measured runtime data were fit with bivariate quadratic functions to develop models for predicting runtime given input dataset and node type. Quadratic functions were chosen as an appropriate polynomial model because a linear function was too simple to fit the data, while a cubic model did not improve performance enough to offset the added complexity. Initial profiling results showed that the measurement data depend on three variables: database size, query size, and node type. The node type variable *k*, which does not change the execution flow, is fixed to model the remaining two-dimensional data with a bivariate polynomial defined in Eq. 8, where $D_i$ represents database size and $Q_j$ represents query size. The coefficient for each term is represented by $c_n$, where *n* is an integer between 1 and 6 corresponding with the six term numbers.

8 $$\begin{aligned} T_k(D_i,Q_j)=c_{1}{D_i}^2+c_{2}{D_i}{Q_j}+c_{3}{Q_j}^2+c_{4}{D_i}+c_{5}{Q_j}+c_{6} \end{aligned}$$

### Load balancing

Performance models can be used to determine the optimal data partitioning of a BLAST job to be processed by multiple nodes on an HPC cluster. Consider a static, centralized, and predicting-the-future load balancing strategy [15]; two cases are discussed in this section.

#### Homogeneous HPC cluster

A homogeneous HPC cluster consists of identical nodes which have the same hardware specifications and are expected to deliver similar performance. Consider a BLAST job of (*D*, *Q*), where *D* and *Q* are two integers representing the total number of sequences for the database and query, respectively, that are concurrently processed by *P* nodes. The database and query can be divided into *m* and *n* fragments, respectively, such that $P=m \times n$. Each node processes a BLAST sub-job of fixed size $(D_i,Q_j)$, where $D_i = D/m$, $1 \le i \le m$, and $Q_j=Q/n$, $1 \le j \le n$.

The optimal solution on a homogeneous node type *k* cluster is the (*m*, *n*) pair which yields the minimum time $T_k(D/m,Q/n)$. The pseudo-code is described below.

**Table:** 

**Algorithm 1:**	
Input: BLAST problem with database size *D* and query size *Q*, to be executed by *P* number of Type *k* nodes, whose performance model is given by *T*	
Output: the optimal (*m*, *n*) that yields the minimum time $T_k(D/m,Q/n)$	
1 Factorize *P* to determine all non-zero integer pairs (*m*, *n*) for which $P=m \times n$.	
2 Evaluate the runtime $T_k(D/m,Q/n)$ for all (*m*, *n*).	
3 Return (*m*, *n*) that minimizes $T_k(D/m,Q/n)$.	

#### Heterogeneous HPC cluster

A heterogeneous HPC cluster consists of non-identical nodes which have different hardware specifications and are expected to deliver different performance. In this case, nodes should process data based on their computing power. Load balancing on a heterogeneous cluster can be solved heuristically with Algorithm 2. The accumulated computing power of each node type is calculated, and the query is divided into sub-parts proportional to the computing power. Since a divide-and-conquer approach is being used, we choose only one dimension to divide–either database or query. Our findings show that dividing the query is more effective, so we decided to divide the query in this algorithm. The identical nodes are then treated as a homogeneous cluster and processed using Algorithm 1.

**Table:** 

**Algorithm 2:**	
Input: BLAST problem with database size *D* and query size *Q*, to be executed by $P_k$ number of Type *k* nodes, whose performance model is given by $T_k$. The performance factor of each node type is given as $s_k$.	
Output: $(m_k, n_k)$ for each node type *k*	
1 For each node type, calculate the accumulated computing power $W_k=s_k \times P_k$.	
2 Divide *Q* into $Q_1$, $Q_2$,..., $Q_k$ such that $\frac{Q_1}{W_1}=\frac{Q_2}{W_2},...=\frac{Q_k}{W_k}$.	
3 For $P_k$ nodes of Type *k*, where $P_k$ is an integer, solve the problem $(D,Q_k)$ as a homogeneous cluster as follows:	
4 Given the total number of database sequences *D*, the number of query sequences $Q_k$, and number of nodes $P_k$ of Type *k*, determine all integer pairs $(m_k,n_k)$ for which $P_k=m_k \times n_k$.	
5 Evaluate the runtime $T_k(D/m_k,Q_k/n_k)$ for each $(m_k,n_k)$ by using the performance models for node type *k*.	
6 Return $(m_k,n_k)$ for the minimum $T_k(D/m_k,Q_k/n_k)$.	
7 Repeat steps 4-6 for each node type until the optimal partitioning for each node type has been determined.	

## Results

### Initial profiling results

The BLAST+ 2.12.0 package contains more than 3,800 source code files written in C++ (e.g., *.cpp and *.hpp). According to the preliminary results from gprof, we found that a specific function, RunMTBySplitDB, in “blastn_app.cpp,” called 53 different functions and constituted more than 99% of the overall execution time and thus needed to be studied in depth.

After analyzing the source code and original comments, we found that the RunMTBySplitDB function can be divided into the following five stages. The inner three stages forms the main loop iterating through all queries. The five most time-consuming functions are annotated in their corresponding stages and are labeled *a* to *e* from greatest to smallest percent overall runtime.

1. Pre-loop
  Get user-specified options (database, query, formatting)
  Initialize database after checking sequence
  Process input (set batch size and target hits)
2. Prepare data
  Prepare data (Function *d*, GetNextSeqBatch)
3. BLAST algorithm
  Create objects (Function *b*, CLocalBlast::CLocalBlast)
  Run BLAST (Function *c*, CLocalBlast::Run)
4. Formatting output
  Prefetch sequence data (Function *e*, PreFetchSequenceData)
  Format and output results (Function *a*, PrintOneResultSet)
5. Post-loop
  Print epilogue and dump debug text
  Format and log BLAST search information

Table 2 shows the shell-, code-, and system-level profiling results against the code structure of the BLASTN application for a 16k database and a 54k query on Type II nodes with Query A. By shell-level profiling, the BLASTN runtime was measured as 135.72 s. By code-level profiling, 99.92% of the BLASTN runtime was found to be due to the function RunMTBySplitDB. After inserting five stopwatches to divide RunMTBySplitDB into five stages, the inner three stages were found to occupy 97.11% of the total BLASTN runtime. Finally, using system-level time profiling with gprof, the top five functions called by RunMTBySplitDB were identified. These five functions make up 92.12% of the total BLASTN runtime. The call counts of the five core functions are also listed in the table. Function *a*’s call count was the same as the query size, while the other four functions were all called four times each. We noted that a significant portion of the runtime was attributed to I/O operations, such as formatting the output which takes up one-third to one-half of the time spent in RunMTBySplitDB. Because BLAST dumps all results into a single output file, runtime of such serial output operations heavily depends on the storage device configuration (e.g., hard disk drive, solid state drive, RAM disk, and memory caching), and cannot be improved by re-distributing the input datasets without modifying the original BLAST workflow.

**Table 2:** Profiling results for a BLASTN job of (16k, 54k)

Unix time (s)	BLAST PROF (s)	BLAST PROF (%)		Unix gprof (%, # calls)		
BLASTN 135.72	RunMTBySplitDB 135.60	#1 Pre-loop	0.02%			
		#2 Prepare data	10.91%	d) GetNextSeqBatch	9.72%	4
		#3 BLAST algorithm	38.04%	b) CLocalBlast	29.28%	4
				c) CLocalBlast::Run	17.00%	4
		#4 Formatting output	48.13%	e) PreFetchSequenceData	5.46%	4
				a) PrintOneResultSet	30.66%	53555
		#5 Post-loop	0.01%			
100%	99.12%		97.11%		92.12%	

### Profiling data analysis

#### Overall Runtime vs Database Size, Query A Size, and Node Type

**Fig. 2:**
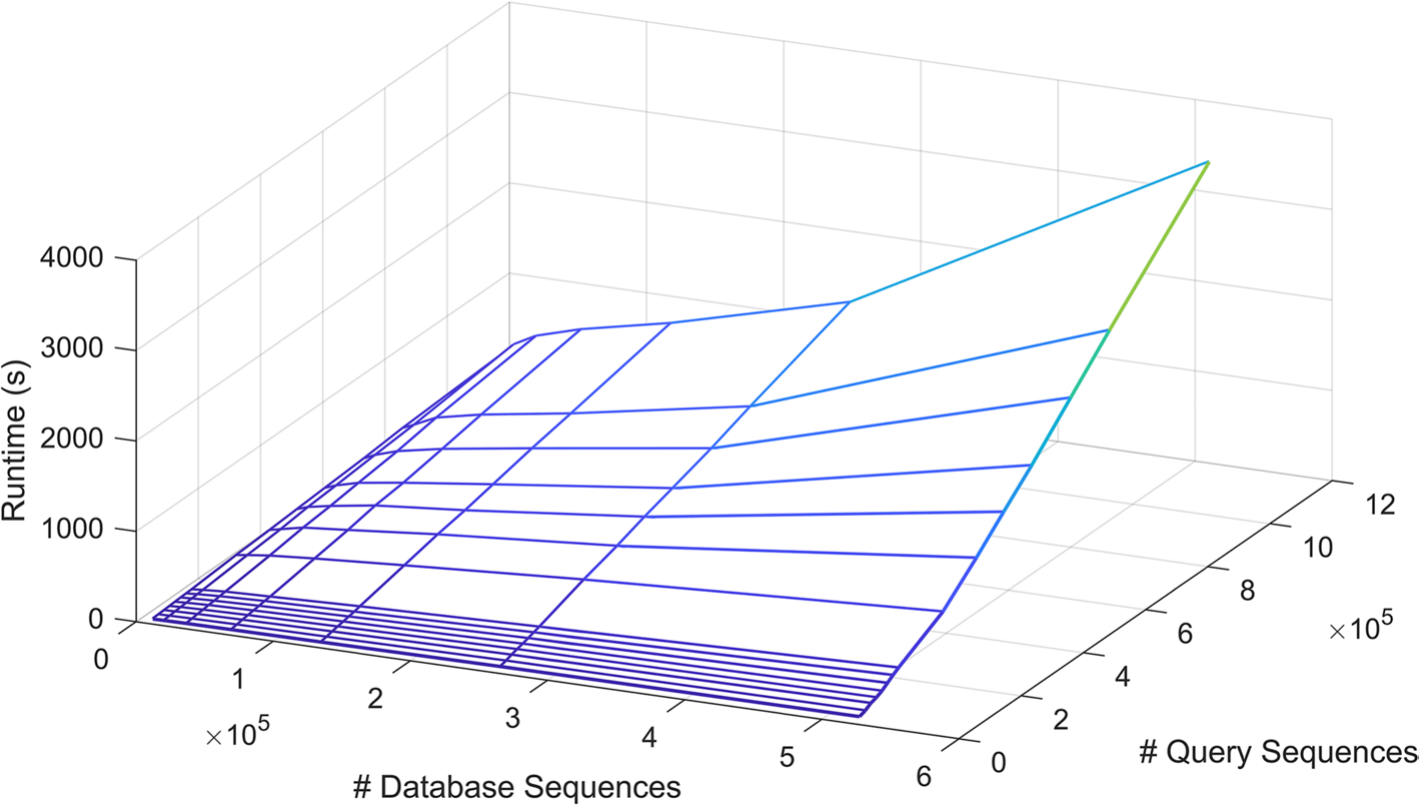
Overall BLASTN runtime vs. database size and Query A size on Type IV nodes, collected by shell-level profiling

**Fig. 3:**
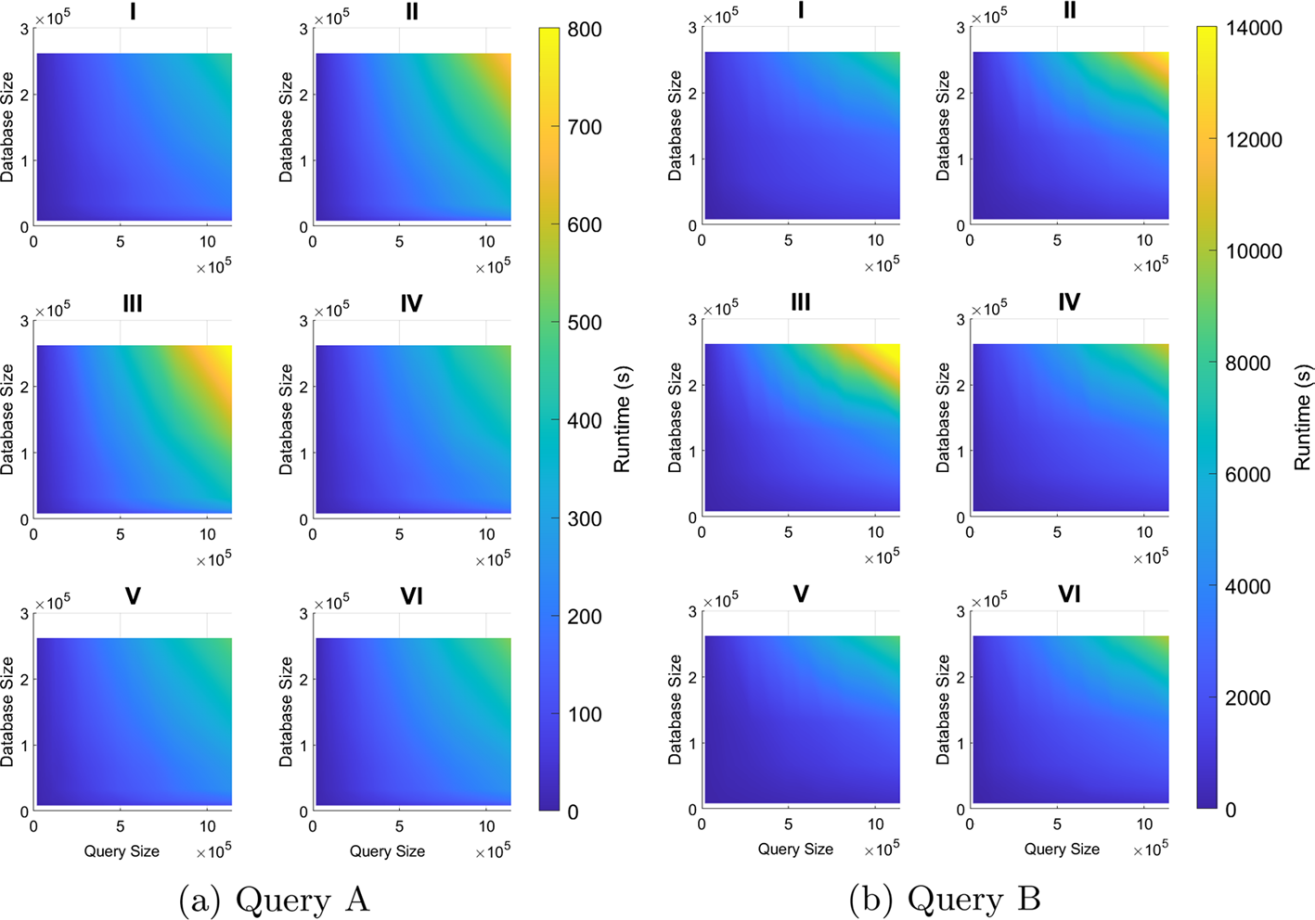
Runtimes (in seconds) of six different node types for different database and query size combinations

The overall runtimes of BLASTN collected by shell-level profiling on Type IV nodes, $T_{IV}(D_i,Q_j)$, are shown in Fig. 2. The blue mesh of the 90 datapoints angles downwards with the lowest point near (0,0). As expected, runtimes decrease monotonically as query size and database size decrease. However, this surface descends faster in the query dimension, indicating that reducing the query size is more performance-effective than reducing the database size. Figure 3a shows the runtimes of six different node types as heatmaps.

The same experiments were repeated using Query B. The runtimes of the six different node types are shown in Fig. 3b. Again, runtimes decrease monotonically with decreasing query size and database size. The absolute runtimes of the Query B are around twice as large as those of the Query A. However, the shape of the surface, and thus the relationship between runtime and database/query size, is still quite similar across the two different queries investigated in this study.

#### Overall runtime vs. multithreading

**Fig. 4:**
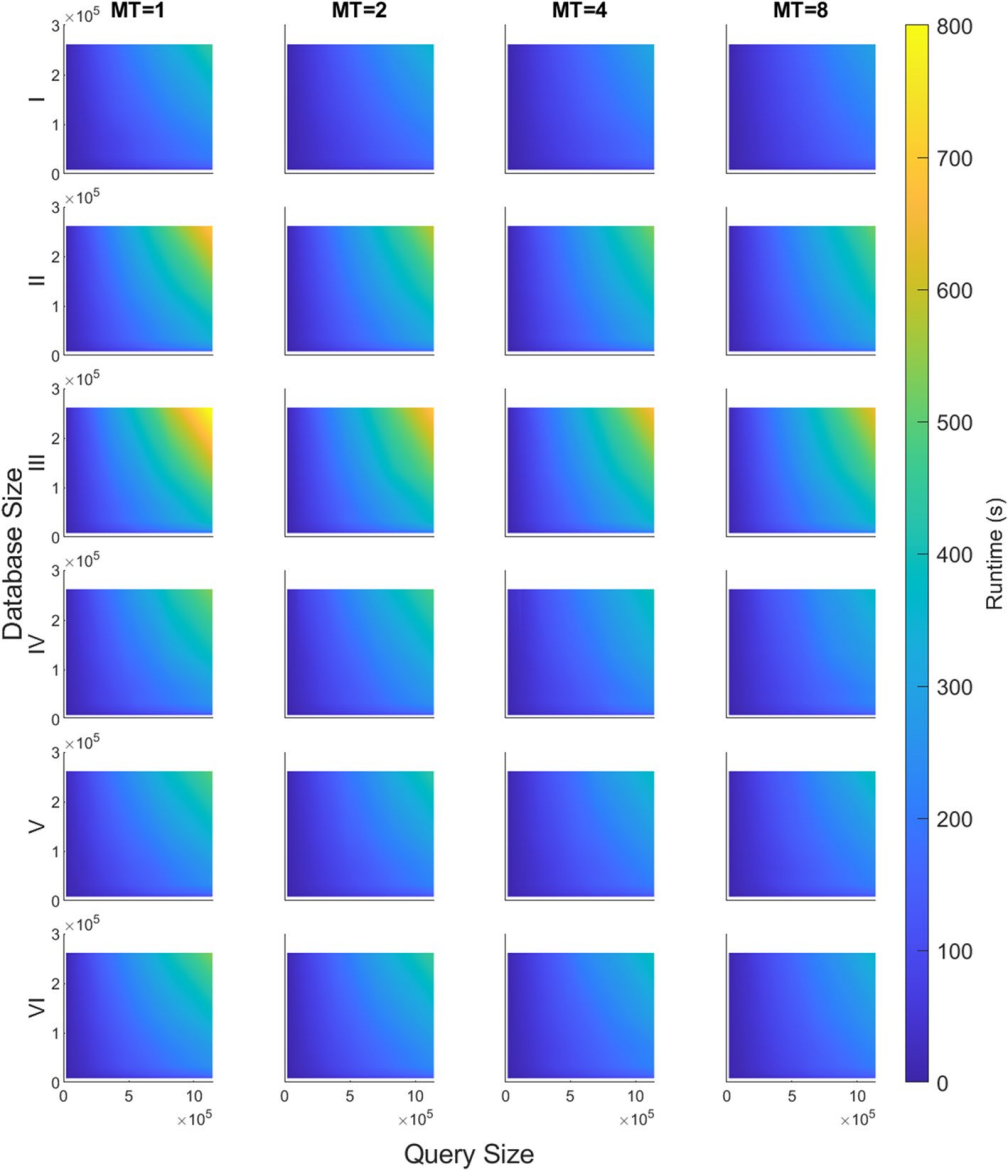
Scalability of multithreading. Runtimes (in seconds) of six different node types for different numbers of threads, using Query A. MT, or multithreading, represents the number of threads used. Each row represents a node type

The overall BLASTN runtimes with multithreading enabled were collected by shell-level profiling on the six node types. The 90 combinations of database and query were run with two, four, and eight threads for each of three trials, using both queries. Figure 4 shows the average runtime collected for Query A as heatmaps. The columns represent the number of threads and the rows represent node type. As expected, all runtimes decrease as more threads are used to simultaneously process the data. However, the shape of each surface remains the same, indicating that the relationship between database size, query size, and runtime does not change with different numbers of threads. While not shown in Fig. 4, the same tests were conducted with Query B, with runtimes across different numbers of threads showing similar trends.

Multithreading with two, four, and eight threads resulted in improvements in runtime for both Query A and Query B. Table 3 shows the speed-up for different multithreading configurations with Query A and Query B. Each column represents a different number of threads. Runtimes were averaged across the 90 database-query combinations and the six node types for each multithreading configuration. Speed-up was calculated relative to single-threading BLAST runtime.

For both datasets, using two threads instead of one resulted in the biggest improvement, compared to going from two to four or four to eight threads. Multithreading improved the runtime of Query B more than for Query A, for all configurations of multithreading.

Multithreading speed-up seemed to slow down as the number of threads was increased from four to eight. Because additional threads did not reduce runtime efficiently, it seems BLASTN may not be a CPU-bound but an I/O-bound application. This observation is also supported by our system-level profiling data as shown in Initial Profiling Results. A majority of BLASTN runtime is spent fetching input data and writing results into the output file (e.g., function *a*).

**Table 3:** Normalized speed-up of multithreading, relative to runtime of single-threading

Query	Thread number			
	1	2	4	8
A	1.00	1.12	1.20	1.24
B	1.00	1.25	1.40	1.53

#### Function runtime vs. database size, query size, and node type

**Fig. 5:**
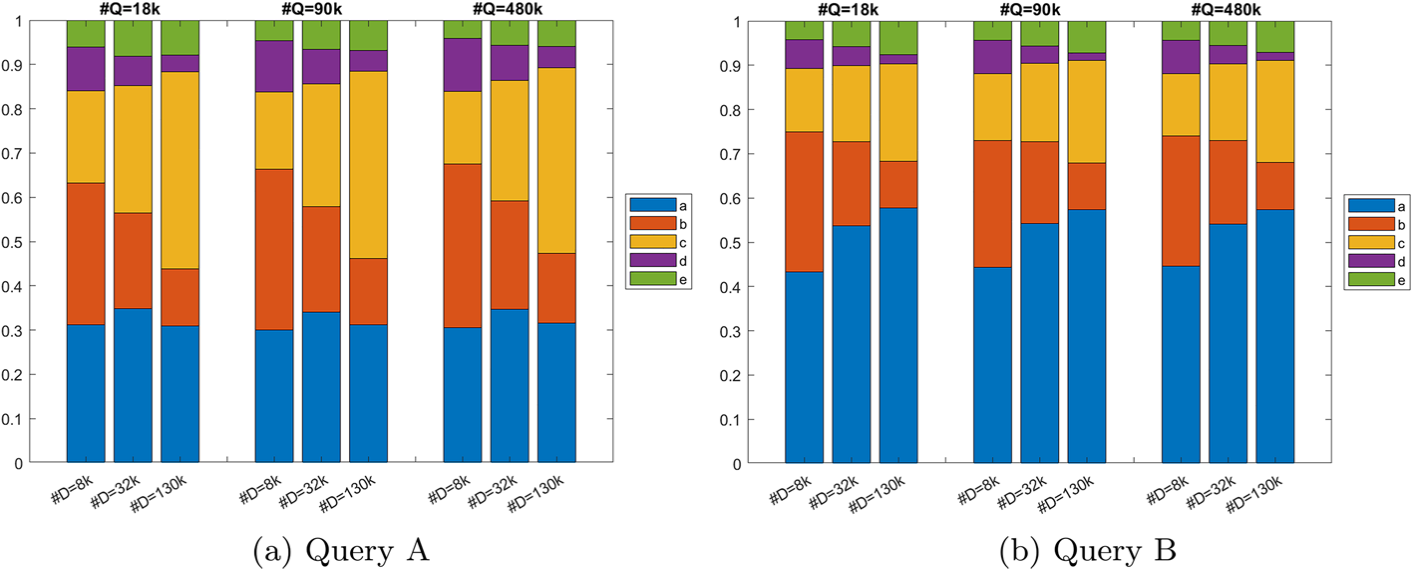
Distributions of function runtimes measured by system-level profiling on Type I nodes for nine database-query combinations. Each group of bars represents one query size (*Q*) while the three bars within each group represent three different database sizes (*D*). The height of each bar represents percentage

Runtimes of the five core functions were collected by system-level profiling. These overall runtimes sum the runtimes of all calls to the same function. Figure 5a shows the distribution of function runtimes for nine database-query combinations run on Type I nodes with Query A. It is observed that the distribution of function runtime stays relatively constant with changing query size. However, as database size changes, function runtime changes as well. With the 8k database, function *a* takes the largest proportion of time. However, as database size increases to 130k sequences, function *c* takes the most time, with a runtime greater than the other four functions combined.

System-level profiling was also conducted to evaluate the runtime distribution of Query B. Even with different sequence content, similar patterns were observed. Figure 5b shows the distribution of function runtimes for nine database-query combinations run on Type I nodes, using Query B. With both queries, functions *b* and *d* decreases in runtime as database size increased. Additionally, the runtime of function *c* increases with increasing database size in both queries. One difference is that as database size increases to 130k sequences, function *a* takes the most time for Query B, with a runtime greater than the other four functions combined. For Query A, the runtime of function *a* seems to stay relatively constant with changing database size.

#### Normalized function runtime vs. query size

**Fig. 6:**
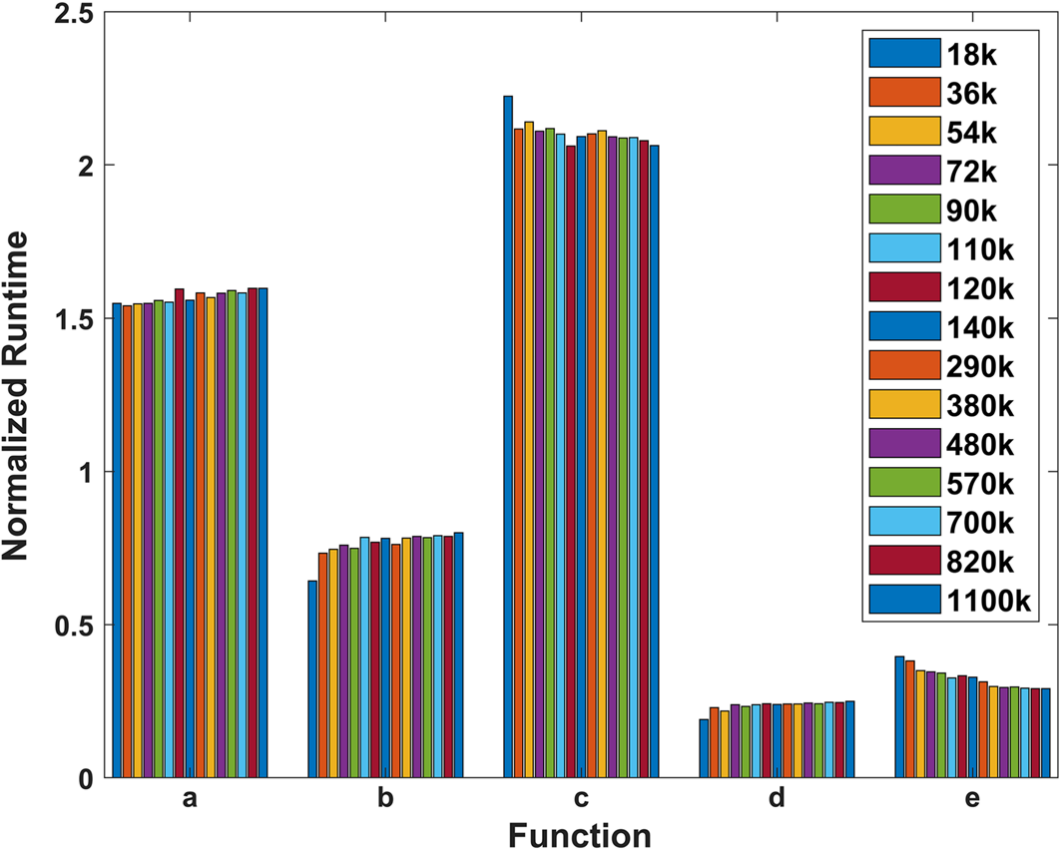
Normalized function runtimes with respect to the mean vs. Query A size. The 15 colors represent different query sizes with database size fixed at 160k sequences. The means were calculated by averaging the five function runtimes for each of the 15 query sizes

Runtimes of the five core functions were measured for 15 different query sizes on Type I nodes using Query A. The database size was fixed at 130k sequences. The runtimes were normalized with respect to the mean and plotted in Fig. 6. The results suggest that query size had little impact on normalized function runtime.

#### Normalized function runtime vs. database size

**Fig. 7:**
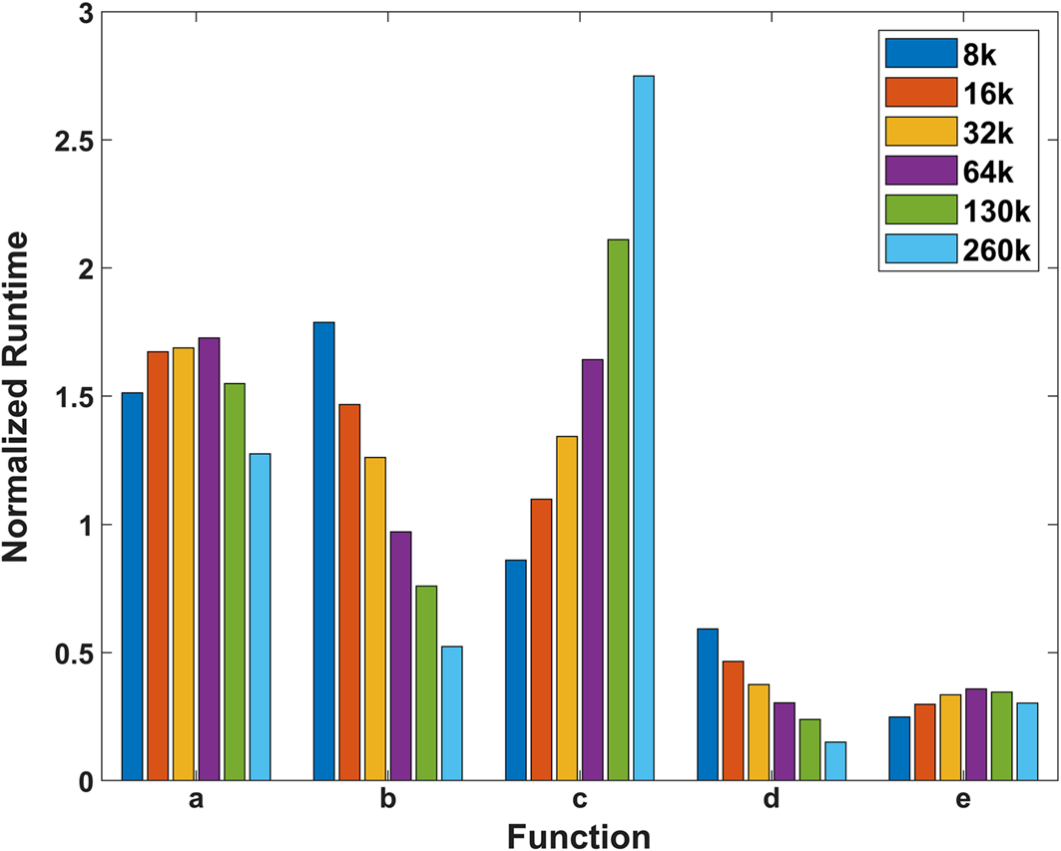
Normalized function runtimes with respect to the mean vs. database size. The six colors represent different database sizes with Query A size fixed at 72k sequences. The runtimes of the five functions were averaged for each database size

Runtimes of the five core functions were measured for six different database sizes on Type I nodes, with Query A size fixed at 72k sequences. The runtimes were normalized with respect to the mean and plotted in Fig. 7. The results show no identifiable trend between normalized function runtime and database size.

**Table 4:** Normalized function runtimes of six node types relative to Type V nodes.

Function	Node type					
	I	II	III	IV	V	VI
*a*	0.7301	1.4077	1.5865	1.0775	1	1.0018
*b*	0.7941	1.3814	1.4713	1.0833	1	1.0912
*c*	0.8018	1.4704	1.5186	1.0689	1	1.1191
*d*	0.6942	1.4758	1.6585	1.0806	1	0.9484
*e*	0.7163	1.4249	1.5763	1.0743	1	0.9910

Based on this table, we decide to use the Constant Performance Model. The $s_i$ of each node type can be determined

#### Function runtime vs. node type

**Fig. 8:**
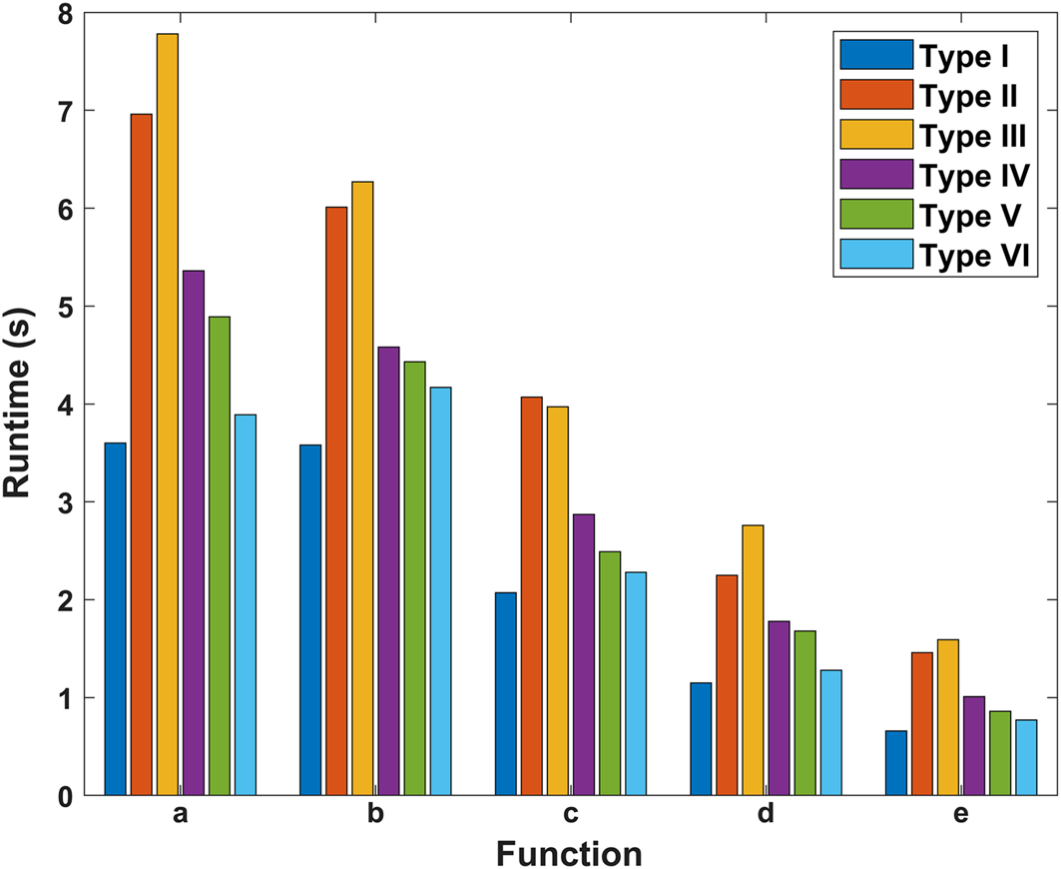
Function runtimes of six different node types

**Fig. 9:**
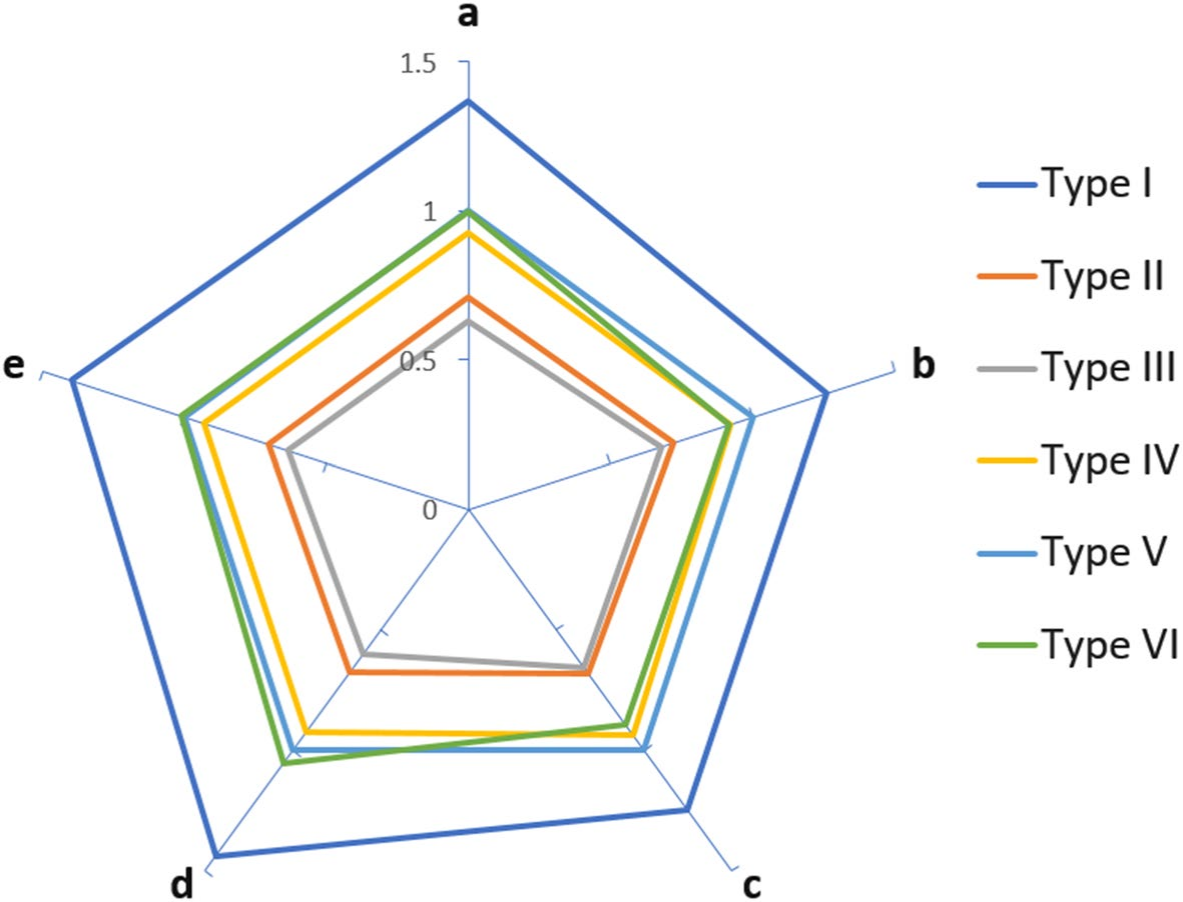
Normalized performance of six node types with respect to Type V nodes for the five core functions

Runtimes of the five core functions were measured for the six different node types using Query A and plotted in Fig. 8. The database size and query size were fixed at 8k and 18k sequences, respectively. The *x*-axis represents functions *a*, *b*, *c*, *d*, and *e*, and the *y*-axis represents the runtime in seconds. The six different lines correspond to six different node types, showing that different node types delivered different performance – Type I the fastest and Type III the slowest. The performance can be sorted in the following order: I> VI> V> IV> II> III, except for two datapoints on function *c*. Among the six node types, the runtime ratio between the five functions has a similar trend but no definitive pattern was observed. Based on the concept of abstract processor [16], the normalized function runtimes with respect to node type are listed in Table 4 and Fig. 9 provides a more intuitive view of the relative performance of the six node types.

### Performance modeling

#### Modeling function runtime

**Fig. 10:**
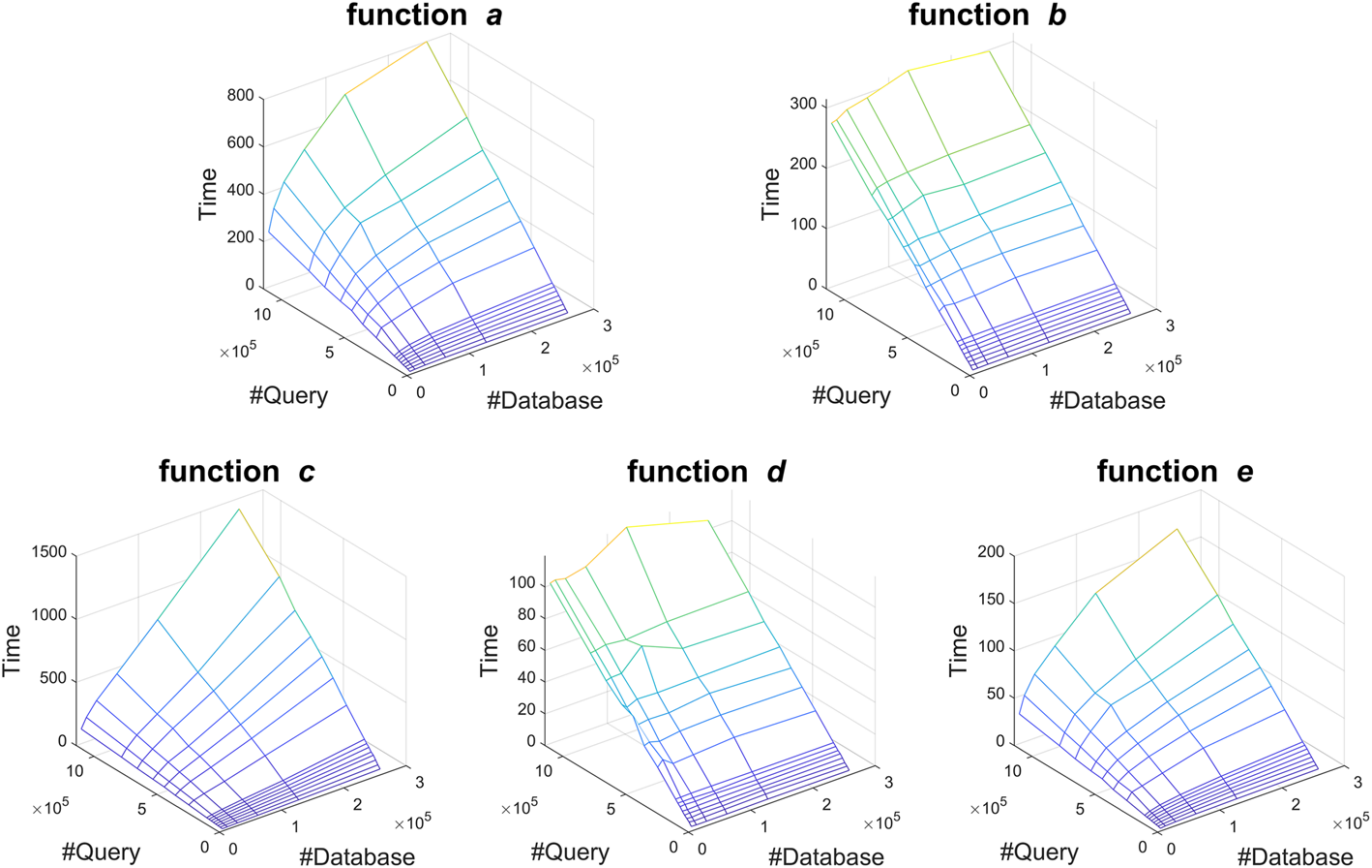
Runtimes of the five core functions vs. database size and query size. Runtimes of five core functions vs. database size (*x*-axis) and query size (*y*-axis) on Type V nodes using Query A. The surfaces of functions *a* and *e* are slightly domed, while those for functions *b*, *c*, and *d* are planes. Notice that for functions *a*, *b*, *d*, and *e*, the surface descends faster in the query dimension, indicating that reducing query size has a greater effect on runtime

The function runtimes were plotted as 3D surfaces in Fig. 10. Functions *a* and *e* have a similar, domed shape. Functions *b* and *d* resemble planes which are proportional to the query size. Function *c* can also be modeled by a plane, which is proportional to both query size and database size.

**Fig. 11:**
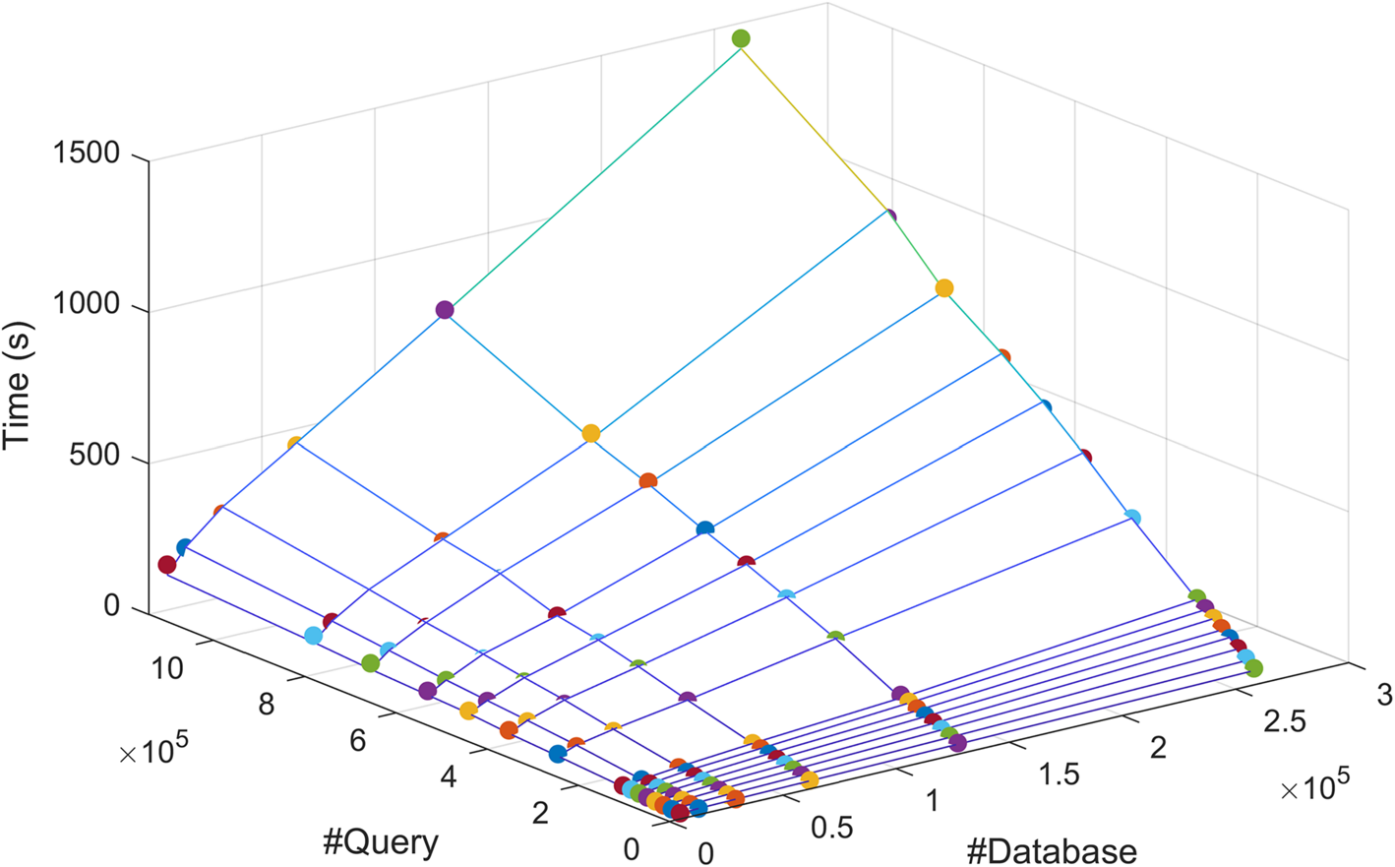
Fitting function *c* with a two-variable linear equation. The 3D mesh represents the fitted equation while the points represent the collected reference data

Multiple linear regression using MATLAB software (The MathWorks Inc., Natick, Massachusetts) was conducted to find the best fit for the profiling data collected with Query A. Table 5 lists the fitting results as the coefficients of Eq. 8. $D_m$ is the number of database sequences in millions and $Q_m$ is the number of query sequences in millions. The zero entries in Table 5 imply that functions *b*, *c*, and *d* are fitted by a plane rather than the domed surface. Additionally, the high ratio of $c_5$ to $c_4$ in functions *a*, *b*, *d*, and *e* shows the greater influence of query size. Figure 11 shows the fitting of one dataset.

After testing different fits, $log(D_m)$ was selected as the input for functions *a* and *e* to achieve the best fit. Since functions *b*, *c*, and *d* are dominated by the query and resemble planes, using *log* did not improve the fit much. For simplicity, $log(D_m)$ was not used as input for those functions.

**Table 5:** Results of using quadratic polynomial Eq. 8 to fit the surfaces in Fig. 10 for $D_m$ database sequences and $Q_m$ query sequences on Type V nodes.

Function	Input		Coefficients					
	*x*	*y*	$\user2{c}_{1}$	$\user2{c}_{2}$	$\user2{c}_{3}$	$\user2{c}_{4}$	$\user2{c}_{5}$	$\user2{c}_{6}$
			($x^2$)	(*xy*)	($y^2$)	(*x*)	(*y*)	(1)
*a*	$log(D_m)$	$Q_m$	−3.0722	143.6097	3.7202	22.4770	881.6166	30.0805
*b*	$D_m$	$Q_m$	0	92.8758	0	0.1523	244.7267	0.4292
*c*	$D_m$	$Q_m$	0	4,429.4587	0	57.7969	94.9201	0.1854
*d*	$D_m$	$Q_m$	0	19.2608	0	−2.6282	90.1090	0.2345
*e*	$log(D_m)$	$Q_m$	−2.0973	33.3526	2.0294	12.8749	181.6523	18.8566

Functions *a* and *e* are saddle-like, but functions *b*, *c* and *d* degenerated to planes due to the zero coefficients

#### Modeling node performance

Different node types take varying amounts of time to run the same function. However, the relationship between function runtime and database/query size is similar across all nodes types as shown in Table 4. In this study, these relative runtimes are used as the scaling factors to estimate the runtimes between different node types.

### Load balancing experiments

Four use cases were tested for a BLASTN job $(D,Q)=(523449, 73102023)$. The experiments were conducted on an FDA HPC cluster with 2,048 and 4,096 nodes. Two node types (II and V) were used to configure homogeneous vs. heterogeneous HPC clusters. The Son of Grid Engine (SGE) system was used to submit the BLAST sub-jobs. The runtimes of the first sub-job are reported and compared in this section.

**Fig. 12:**
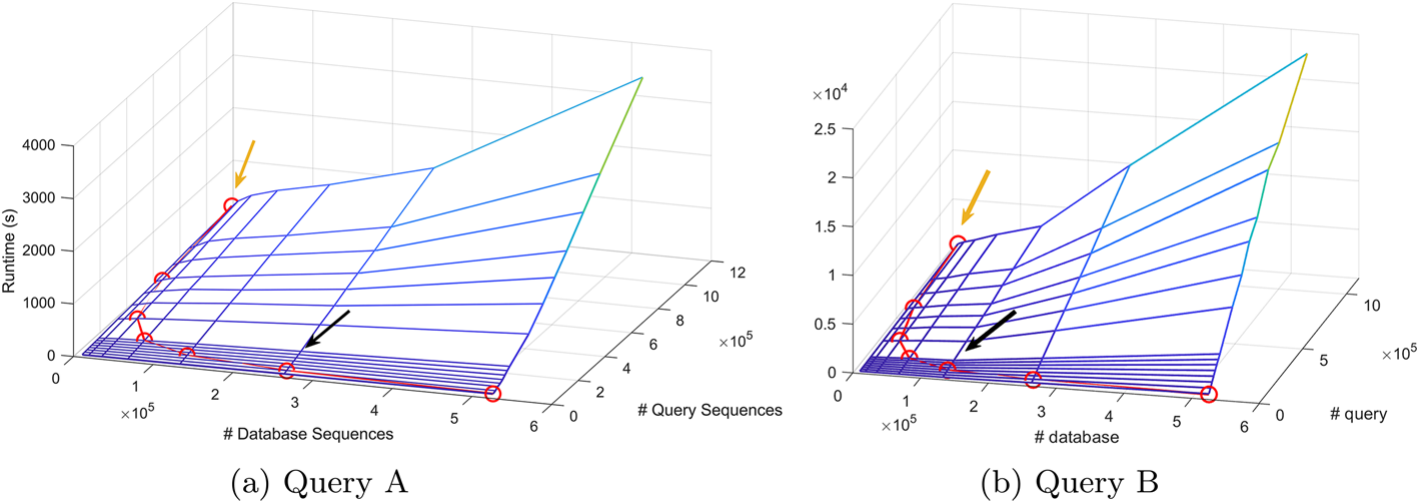
BLASTN runtime vs. database and query size for different queries. BLASTN runtime on Type V nodes (*z*-axis) vs. database size (*x*-axis) and query size (*y*-axis). The blue mesh represents the measured runtime. The red hyperbolic curve represents the possible runtimes of an HPC system with 4096 Type V nodes. The orange and black arrows represent the results from Test Cases 1 and 2, respectively

Table 6 lists the candidate database-query splits (*m*, *n*) and their corresponding sub-job sizes $(D_i,Q_j)=(D/m,Q/n)$. Each $(D_i,Q_j)$ can be represented as a point located on a two-dimensional hyperbolic curve on the x-y plane, as seen by the red curves in Fig. 12. This two-dimensional hyperbolic curve can be projected onto the three-dimensional surface, constructed by the runtime estimate for Type V nodes (blue meshes in Fig. 12), to define our solution space.

**Table 6:** Different combinations of *D*/*m* and *Q*/*n* for *D*=523, 449, *Q*=73, 102, 023, and *P*=4, 096

*m*	*n*	$\user2{m} \times \user2{n}$	$\user2{D}_{i} = \user2{D}/\user2{m}$	$\user2{Q}_{j} = \user2{Q}/\user2{n}$
1	4,096	4096	523,449	17,848
2	2,048	4096	261,725	35,695
3	1,365	4095	174,483	53,555
4	1,024	4096	130,862	71,389
5	819	4095	104,690	89,258
6	682	4092	87,242	107,188
7	585	4095	74,779	124,961
8	512	4096	65,432	142,778
16	256	4096	32,716	285,555
32	128	4096	16,358	571,110
64	64	4096	8,179	1,142,220

**Test Case 1:** The BLASTN job was distributed on a homogeneous cluster of $4096=m \times n$ Type V nodes, where *m* and *n* were chosen to be close as $m=n=\sqrt{P}=\sqrt{4096}=64$ based on the approach used in [12]. Each node ran a sub-job $(D_i,Q_j)$, where $D_i=D/m=8179$ sequences and $Q_j=Q/n=1,142,220$ sequences, represented by the point (8179, 1142220), indicated by the orange arrow on the red hyperbolic curve in Fig. 12a. The predicted runtime was 242.74 s, and the actual runtime of the first sub-job was 204.15 s.

**Test Case 2:** The BLASTN job was distributed on a homogeneous cluster of $4096=m \times n$ Type V nodes, where *m* and *n* were chosen using Algorithm 1 as $(m,n)=(2,2048)$, which recommends minimizing the query size rather than the database size. This minimum $T_V(D_i,Q_j)$ is the lowest point on the red hyperbolic curve in Fig. 12a, representing the optimal solution with the shortest runtime. Each node ran a sub-job of $(D/m,Q/n)=(261725,25695)$, indicated by the black arrow in Figure 12a. The actual runtime of the first sub-job was 37.79 s, which was 5.4 times faster than Test Case 1. In comparison, the predicted runtime was 49.82 s, which was 4.9 times faster than Test Case 1.

**Test Case 3:** The BLASTN job was distributed on a heterogeneous cluster of 2048 nodes of Type II and 2048 nodes of Type V. The job was partitioned using the same method as in Test Case 2, i.e., $(m,n)=(2,2048)$, without considering the performance differences between nodes. Each node, regardless Type II or Type V, ran a sub-job of (261725, 25695). The actual runtime of the first sub-job was 58.52 s on Type II nodes, but only 37.79 s on Type V nodes. In comparison, the predicted runtime was 64.61 s on Type II nodes, and 49.82 s on Type V nodes.

**Test Case 4:** The BLASTN job was distributed on a heterogeneous cluster of 2048 nodes of Type II and 2048 nodes of Type V. The job was partitioned into two parts to balance the load between two node types. By using Algorithm 2, the recommended partitioning was $Q_{II}$=26, 624, 000 and $Q_V$=46, 479, 360. Type V nodes processed a greater portion of data, 63.58%, as opposed to 50% as in Test Case 3. When $P_{II}$=2, 048 and $P_V$=2, 048, the Type II nodes processed a query subset of $Q_{II}/P_{II}=13,000$ sequences while the Type V nodes processed a query subset of $Q_V/P_V=22,695$ sequences. The actual runtime of the first sub-job was 46.89 for Type II nodes and 46.97 s for Type V nodes, resulting in 19.94% less runtime than test case 3. In comparison, the predicted runtime was 80.72 s for Type II nodes and 77.93 for Type V nodes, resulting in 19.96% less runtime than Test Case 3.

The results from the four test cases are summarized in Table 7.

**Table 7:** Runtimes on homogeneous and heterogeneous HPC clusters

Test case	Node type II		Node type V	
	*(D/m,Q/n)*	Time (s)	*(D/m,Q/n)*	Time (s)
	$\times$ # nodes		${\mathbf{ \times }}$ # nodes	
1	–	–	(8179,1142220)	204.15
			$\times$ 4,096	
2	–	–	(261725,35695)	37.79
			$\times$ 4,096	
3	(261725,35695)	58.52	(261725,35695)	37.79
	$\times$ 2,048		$\times$ 2,048	
4	(523449,13000)	46.89	(523449,22695)	46.97
	$\times$ 2,048		$\times$ 2,048	

#### Query dependency

Test Cases 1 and 2 were run with Query B. Using the same HPC set-up and data partitioning as Test Case 1, each node again ran a sub-job of (8179, 1142220), indicated by the orange arrow on the red hyperbolic curve in Figure 12b. The runtime of the first sub-job was 405.23 s.

Following the steps outlined in Algorithm 1, *m* and *n* were chosen as $(m,n)=(4,1024)$. Each node ran a sub-job of $(D/m,Q/n)=(130862,71389)$, indicated by the black arrow in Figure 12b. The actual runtime of the first sub-job was 156.29 s, which was 2.6 times faster than Test Case 1.

#### Multithreading

Test Cases 1 and 2 were also repeated with multithreading enabled. Figure 14 shows the points that are in the solution space of an HPC system with 4,096 nodes. Each line represents a different number of threads. Using an increasing number of threads resulted in decreasing absolute runtimes, but the relative runtime remained the same. As a result, the recommended partitioning remains the same even with varying numbers of threads.

**Fig. 13:**
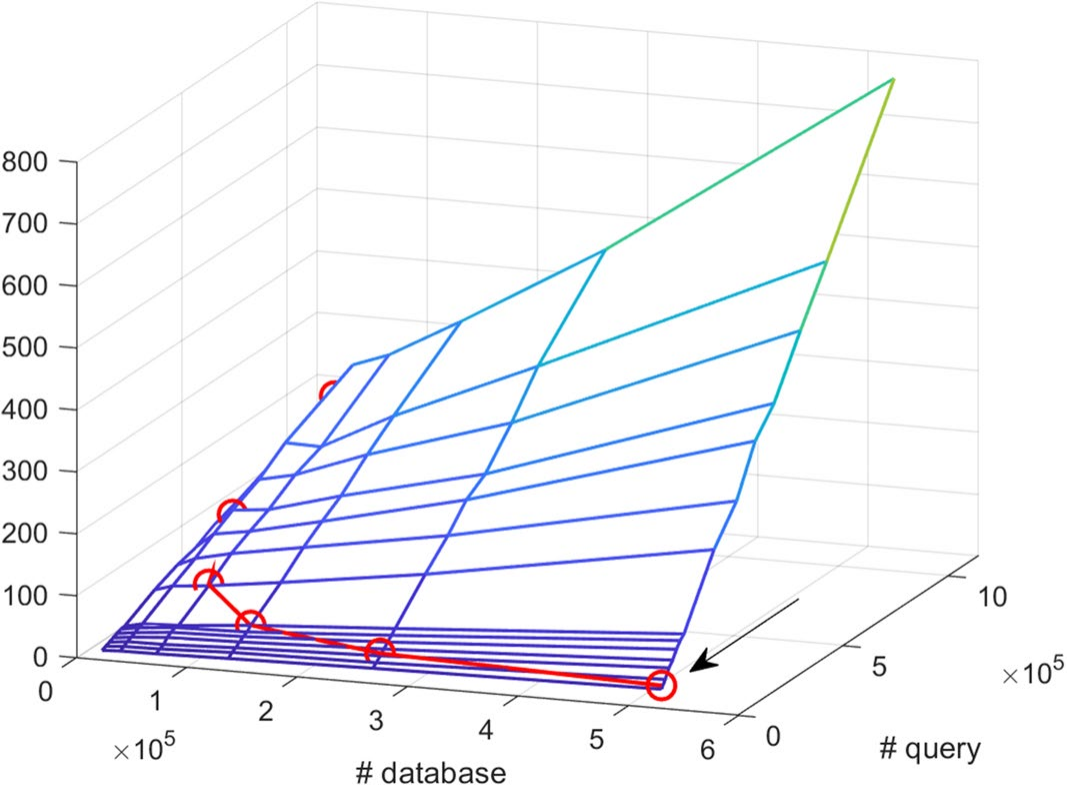
BLASTN runtime with Query A vs. database and query size for an HPC cluster of 2,048 Type III nodes run with a single thread. The blue mesh represents the measured runtime, and the red hyperbolic curve represents the possible runtimes of an HPC system with 2,048 nodes. The black arrow represents the recommended partitioning

#### Node number dependency

The algorithms were tested in a solution space of a homogeneous HPC cluster with 2,048 Type III nodes, using Query A. Table 8 lists database-query splits (*m*, *n*) and their corresponding sub-job sizes $(D_i,Q_j)=(D/m,Q/n)$, when $m \times n = 2,048$.

The $(D_i,Q_j)$ combinations are represented as points located on the red hyperbolic curve in Figure 13. The recommended partitioning was found to be $(m,n)=(1,2048)$, where each nodes runs a sub-job of $(D/m,Q/n)=(523449,35695)$. The runtime for the first sub-job was 48.62 s, indicated by the black arrow in Figure 13. Compared to a more-balanced partitioning of $(m,n)=(32,64)$, which results in a runtime of 298.8 s for the first job, the recommended partitioning is more than six times faster.

**Table 8:** Different combinations of *D*/*m* and *Q*/*n* for *D*=523, 449, *Q*=73, 102, 023, and *P*=2, 048

*m*	*n*	$\user2{m} \times \user2{n}$	$\user2{D}_{i} = \user2{D}/\user2{m}$	$\user2{Q}_{j} = \user2{Q}/\user2{n}$
1	2,048	2,048	523,449	35,695
2	1,024	2,048	261,725	71,389
3	682	2,046	174,483	107,188
4	512	2,048	130,862	142,778
8	256	2,048	65,432	285,555
16	128	2,048	32,716	571,110
32	64	2,048	16,358	1,142,220

**Fig. 14:**
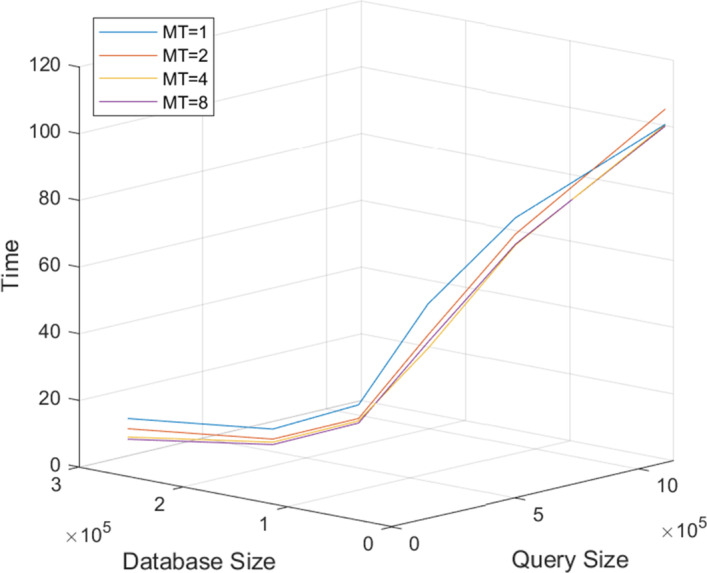
BLASTN runtime vs. multithreading for 4,096 nodes. BLASTN runtime on Type I nodes (*z*-axis) vs. database size (*x*-axis) and Query A size (*y*-axis). The four lines represent the runtimes with multithreading using one, two, four, and eight threads. Each line shows the possible runtimes of an HPC system with 4,096 Type I nodes

## Discussion

In this study, only BLASTN was examined; however, the same principles of profiling, modeling, and optimizing can be applied to other programs and HPC systems. The proposed methodology can be used in the other applications in the BLAST+ suite or any other application as long as source code is provided, as well as with different input files and hardware.

The system-level profiler “gprof” used to identify core functions is powerful but time-consuming to generate and analyze, despite only needing to be done once to create the performance model. Additionally, the measured runtime may be distorted because of the profiling overhead. The model accuracy may depend on other factors such as runtime workload, cache mechanism for the file system, job schedulers, etc. in the HPC cluster.

In this study, BLASTN’s measured runtime was decomposed into five components that were well-fitted by bi-variate quadratic functions. Other applications may not exhibit the same property. The models can guide parallelization of BLAST jobs by recommending the optimal data partitioning on an HPC cluster. In our BLAST problem, users should generally focus on allocating a smaller query to each node rather than a smaller database, as results show that reducing query size is more effective than reducing database size when looking to reduce runtime. Experiment data show that the optimal data partitioning improves overall runtime 5.4-fold in comparison with dividing the database and query into the same number of fragments.

### Limitations

#### Study design

Our method is limited to static load balancing only, in consideration of the actual BLAST use cases where the end user has no real-time control after the input datasets are partitioned and dispatched for execution through the HPC scheduler. Dynamic load balancing requires real-time performance monitoring and data migration controlled by synchronization points. The former is considered a general HPC problem that is beyond the scope of our study. The latter will be addressed by extending our proposed algorithms for future work.

#### Scalability

The scalability of BLAST with respect to number of nodes was not included in the present study, because our research problem was formulated as an optimization problem to utilize all available nodes provided by the user. The user can use our performance model to predict the runtime when a certain number of nodes are available, and determine how many nodes to allocate.

#### Sequence length

The proposed algorithms partition the databases based on their sequence counts only but not their sequence lengths. Although the sequence length is another factor that influences runtime, retrieving and processing this information is time-consuming and may offset the benefits gained from a better partitioning in practice. Retrieving the sequence length requires parsing the file format, in our case the FASTA file format, to count characters in a sequence that may span multiple lines after identifying the delimiter and optional comments. The quantity of collected sequence lengths (e.g., 73 million for Query A and 520 thousand for the database in our study) would become considerable additional workload. Therefore, in the study, we assume that the user has no a priori knowledge about the input databases except for the sequence counts.

#### Sequence content

We found that BLAST runtimes also depend on the sequence content, as previous studies have shown [18]. Sequences of the same length can be processed with a time difference, ranging from a couple of seconds to thousands of seconds, with the results from our study showing a more than 5x difference in time between the two queries evaluated. Based on these observations, it seems that BLAST runtime depends not just on database length, query length, and hardware, but also the underlying content of the database and query.

#### Modeling

Profiling data was collected in the modeling stage and tests in the prediction stage were conducted at different times, where our HPC cluster experienced different workload ordered by the other users. As a result, the performance models have limited accuracy. We can mathematically model the profiling data accurately in the modeling stage. However, the model may not predict the BLAST runtime accurately because the system workload has changed. This is a common problem when the training data deviates from the test data. The solution would be to fix the system workload by grounding all unrelated jobs, which unfortunately is infeasible in our facilities.

## Conclusion

BLAST is one of the most commonly used applications in the bioinformatics field. Due to its computation- and data-intensive nature, BLAST analysis can benefit from parallelization on HPC clusters. However, the optimal data partitioning method for BLAST has not been well-established in the literature. The goal of this study is to develop guidelines for BLAST users to improve performance by partitioning input data optimally. We used three profiling methods to obtain the overall, stage, and function runtimes at shell-, code-, and system-level for the BLASTN application, respectively. Shell- and system-level profiling can be used together to provide valuable runtime information for different use and purposes. Time-consuming system-level profiling can be used for one-time identification of time-consuming functions, while shell-level profiling can be easily utilized to measure BLAST overall runtime for multiple cases. We found that five core functions occupied 92.12% of the execution time and needed to be modeled accurately. Based on the profiling data, unlike the uni-variable performance models proposed in the literature, we modeled the BLASTN runtimes as a three-dimensional function of database size, query size, and node type. We used one database and two queries to demonstrate the utility of our method with four test cases of homogeneous/heterogeneous clusters with different multithreading settings and node numbers. Experiment results show that the runtime can be reduced by 81% on a homogeneous cluster and by 20% on a heterogeneous cluster with node performance differences of 50%.

## Data Availability

The datasets analyzed during this study are included in [12] and are publicly available in the NIH NCBI Nucleotide database, ftp://ftp.ncbi.nlm.nih.gov/blast/db/FASTA/nt.gz and https://trace.ncbi.nlm.nih.gov/Traces/index.html?view=run_browser &acc=SRR5713923 &display=download. Samples of the code used in this study are available at https://github.com/DIDSR/BLAST_LOAD_BALANCING.
